# Bridging Mid- and Near-Infrared by Combining Optomechanics and Self-Mixing

**DOI:** 10.1021/acsphotonics.5c03062

**Published:** 2026-02-06

**Authors:** Tecla Gabbrielli, Chenghong Zhang, Francesco Cappelli, Iacopo Galli, Andrea Ottomaniello, Jérôme Faist, Alessandro Tredicucci, Alessandro Pitanti, Paolo De Natale, Simone Borri, Paolo Vezio

**Affiliations:** † 68229 CNR-INO − Istituto Nazionale di Ottica, Via Carrara, 1, Sesto Fiorentino, Florence 50019, Italy; ‡ 213324 LENS − European Laboratory for Non-Linear Spectroscopy, Via Carrara, 1, Sesto Fiorentino, Florence 50019, Italy; § Center for Materials Interfaces, 121451 Istituto Italiano di Tecnologia, Via R. Piaggio, 34, Pontedera, Pisa 56025, Italy; ∥ Institute for Quantum Electronics, ETH Zürich, Zürich 8093, Switzerland; ⊥ Dipartimento di Fisica, 9310 Universitá di Pisa, Largo B. Pontecorvo 3, Pisa 56127, Italy; # Laboratorio NEST, CNR − Istituto Nanoscienze, Piazza San Silvestro 12, Pisa 56127 Italy; ∇ Dipartimento di Fisica e Astronomia, Università degli Studi di Firenze, Via Sansone 1, Sesto Fiorentino, Florence 50019, Italy

**Keywords:** quantum cascade laser, infrared, feedback interferometry, optomechanics, self-mixing, information transduction

## Abstract

This work describes a self-mixing-assisted optomechanical platform for transferring information between near- and mid-infrared radiation. In particular, the self-mixing signal of a mid-infrared quantum cascade laser is used to detect the oscillation of a membrane driven by light-induced forces exerted by a near-infrared excitation beam, which is amplitude-modulated at the membrane resonance frequency. This technique benefits from spectral broadness and, therefore, can link different spectral regions from both the excitation and the probe sides. This versatility can pave the way for future applications of this self-mixing-assisted optomechanical platform in free-space communication and advanced sensing systems.

## Graphical abstract

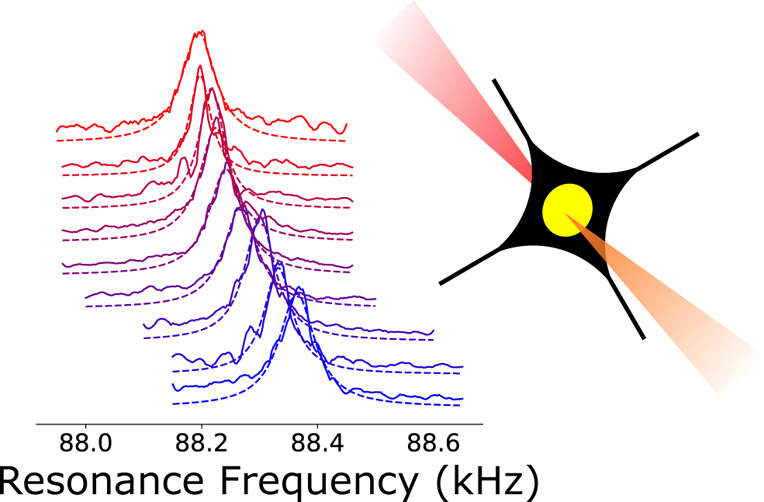

## 1 Introduction

Self-mixing (SM) detection is a homodyne optical feedback interferometry technique where the laser is used both as a source and a detector.^1–3^ In SM-based schemes, the light emitted by the laser source is back-reflected via the target (e.g., a membrane or a mirror) to one of the laser facets. The back-reflected light, reinjected into the laser waveguide, interferes with the intracavity optical field with a phase depending on the target position.^1^ This injection alters the laser’s working point parameters, such as intracavity optical power and laser voltage drop. This technique enables the extraction of target information by directly monitoring the voltage signal measured at the laser terminals^4^ or the laser output power from the other facet.

Quantum cascade lasers (QCLs)^5^ are particularly well-suited to be used in SM setups. Similarly to bipolar semiconductor lasers, they are highly sensitive to optical feedback. On the other hand, since the laser transition takes place between the sublevels of the conduction band created by the semiconductor layers’ heterostructure, the laser transition lifetime is very short (<1 ps).^6^ For this reason, QCLs can be modulated at high speed (GHz and above)^7,8^ and, in principle, are also able to detect SM signals within the same bandwidth. As a consequence, QCLs have been successfully employed for realizing highly integrated sensitive sensors, as feedback-induced variations in laser voltage or output power can carry precise displacement and/or optical path information.^9,10^ Over the years, these SM-based compact sensors have been extensively applied to a wide range of applications, including characterization of laser linewidth and α-factor^11–13^ displacement sensors,^14^ optical detection,^15^ and gas sensing and imaging.^16,17^

Recent studies have explored similar configurations combining QCL self-mixing with suspended membranes, demonstrating the feasibility of hybrid photonic-mechanical platforms.^9,10^ However, these previous studies primarily focused on stationary photothermal effects in systems where membrane motion was piezoelectrically driven and only perturbed by external radiation. In contrast, this work aims to explore a different regime in which dynamic light-induced forces—originating from both radiation pressure and photothermal effects^18^—serve as the primary driving mechanism for membrane oscillation. Specifically, we demonstrate a fully optical actuation scheme in which a trampoline membrane is driven by amplitude modulation of a near-infrared (near-IR) excitation laser and monitored via self-mixing interferometry using a mid-infrared (mid-IR) QCL. This approach enables cryogen-free operation with purely optical actuation and readout, extending functionality to higher mechanical frequencies (≈90 kHz) and shorter detection wavelengths (mid-IR vs THz) compared to previous works.^10^ In other words, we test and demonstrate the ability to encode the membrane oscillation signal, induced by the near-IR excitation beam, in the mid-IR QCL via SM coupling. This means that the membrane, combined with SM, actively enables the transfer of information, i.e., a communication between the two beams at different infrared wavelengths. Moreover, by demonstrating that the light-induced force exerted by the excitation beam is the phenomenon responsible for the membrane oscillation, we prove that this SM-assisted optomechanical platform benefits from being, in principle, broadband in terms of the excitation wavelength.

On one hand, this platform can be used as a communication gate between the mid-IR and other spectral ranges. On the other hand, the universal nature of the SM effect guarantees wavelength independence for the probe source. This further expands the applicability of the proposed platform across most of the frequency spectral range. These results make the presented platform promising for different applications, spanning from communication to sensing and imaging, as discussed in the perspective section (Section 4).

## 2 Experimental Setup and Methodology Description

The experimental setup is shown in Figure 1. The SM signal generated in a Fabry–Pérot QCL emitting at 4.5 μm is used to probe and monitor the optomechanical oscillation induced in a trampoline membrane via a near-IR excitation beam.

**Figure 1:**
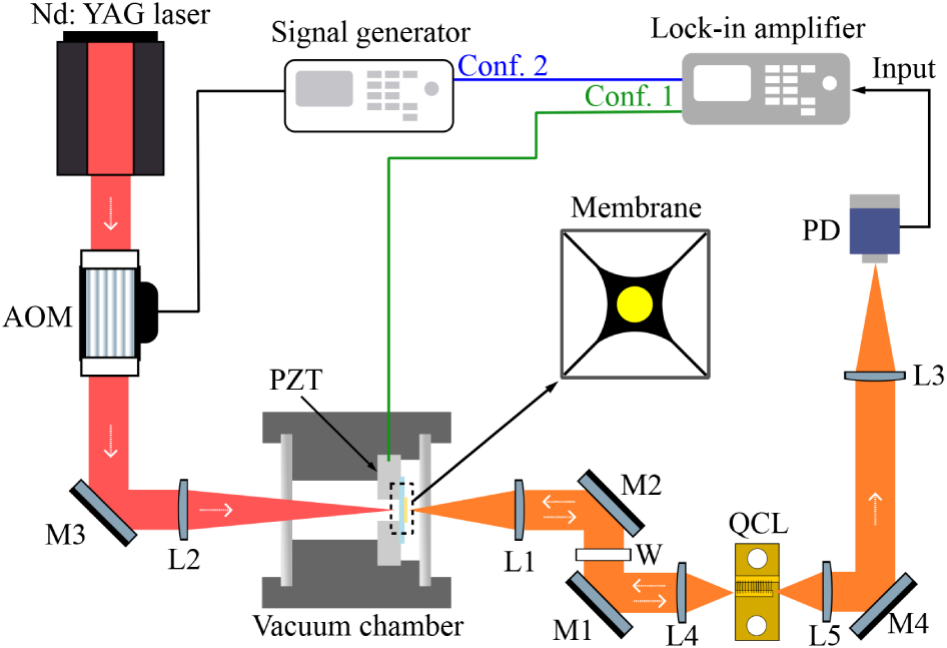
Schematic of the SM setup. M: mirror, W: window, PD: photodetector, L: lens, PZT: piezoelectric actuator; AOM: acousto-optic modulator. The configuration used to characterize the membrane resonance frequency by acting on the piezo, namely, *Conf. 1* is depicted in green. The configuration where the membrane is excited via the AM-modulated near-IR radiation is depicted in blue, namely, *Conf. 2*.

In particular, the trampoline membrane is a four-armed high-stress silicon nitride (Si_3_N_4_) membrane with a metal coating (Cr/Au 5–30 nm coating). The membrane shape is sketched in the center of Figure 1. The triggered mechanical resonance occurs at the fundamental mode. More details about the membrane design and fabrication are available in ref 10. The membrane is fixed on a pass-through-hole piezoelectric actuator (PZT). The PZT can actuate the membrane oscillation, e.g., for calibration purposes. The membrane is housed in a room-temperature vacuum chamber featuring two selected windows that maintain transparency while enabling simultaneous transmission of the impinging laser beams. In detail, a 1-mm-thick cyclic-olefin copolymer optical window is used on the near-IR excitation beam side and a CaF_2_ window on the mid-IR radiation side. The windows are flat to allow reaching a good vacuum in the chamber. The vacuum chamber is pumped at ∼10^–3^ mbar. For alignment purposes, the membrane platform (membrane, vacuum chamber, PZT; see Figure 1) is mounted on a 3-axis stage.

On one side, the membrane is illuminated by the near-IR excitation beam (left side in Figure 1). This beam is obtained by deflecting the radiation of an Nd:YAG laser, emitting at 1064 nm, via an acousto-optic modulator (AOM). The AOM deflects the laser beam and shifts the laser frequency by 245 MHz through a radio frequency (RF) injection. The optical power of the near-IR excitation beam (first order of deflection from the AOM) is controlled by adjusting the RF signal amplitude, and it is focused onto the membrane by using a BK7 75-mm lens (L2) with an antireflection coating at 1064 nm.

On the other side (right side in Figure 1), the membrane is illuminated by the MIR QCL probe beam. The QCL is operated at room temperature (18 °C) in a single-mode regime at a bias current of 470 mA. The laser working conditions are kept constant in all the performed measurements. The QCL mounting arrangement allowed us to conveniently exploit the emission from both laser facets. The front-facet-emitted beam is used to probe the membrane oscillation, receiving back the resulting optical feedback. To this extent, this radiation is focused on the membrane via a CaF_2_ 50-mm lens (L1), and a germanium wedged window (W, window wavelength range 2–16 μm) is used in the probe path to prevent any injection of the near-IR radiation into the QCL. Being uncoated, the lens and window are tilted to avoid back-reflections affecting the stability of the QCL SM response. Instead, the radiation emitted by the back facet of the QCL can be used for the free-space transmission of the MIR radiation carrying the SM signal. This makes the platform exploitable for future mid-IR free-space communication tests in which the back-facet-emitted beam, encoded with the SM signal, can be used as an information carrier. In our setup, after a free-space propagation, the SM signal is retrieved by detecting the back-facet-emitted radiation via a fast commercial photovoltaic detector (PVI-4TE-5-2×2 by Vigo System). The detector output signal is used as theinput signal of a lock-in amplifier. Depending on the application, we highlight that the direct detection of the SM signal from the laser voltage variation could be more convenient as this method requires no extra detector. In particular, the presented optomechanical platform can be conveniently exploited in both ways, whether we are interested in an optical or electrical SM readout.^10^

As depicted in Figure 1, our system can be operated in two different configurations. In the first configuration (*Conf. 1*), the PZT induces membrane oscillation. This configuration is used for system calibration, as it allows us to quantify the photothermal effect on the membrane of both pump and probe under continuous-wave operation (see Section 3). In the second configuration (*Conf. 2*), the membrane oscillation is induced by the AM-modulated excitation beam (without PZT actuation), as discussed in Section 3. In both cases, the lock-in amplifier is used: 1) to control the sinusoidal modulation signal and its frequency sweep, which is sent to the PZT in *Conf. 1* or to the AOM in *Conf. 2*; 2) to demodulate the self-mixing signal measured via the photodetector. This allows the retrieval of the membrane spectral response (e.g., as shown in Figure 2a).

**Figure 2:**
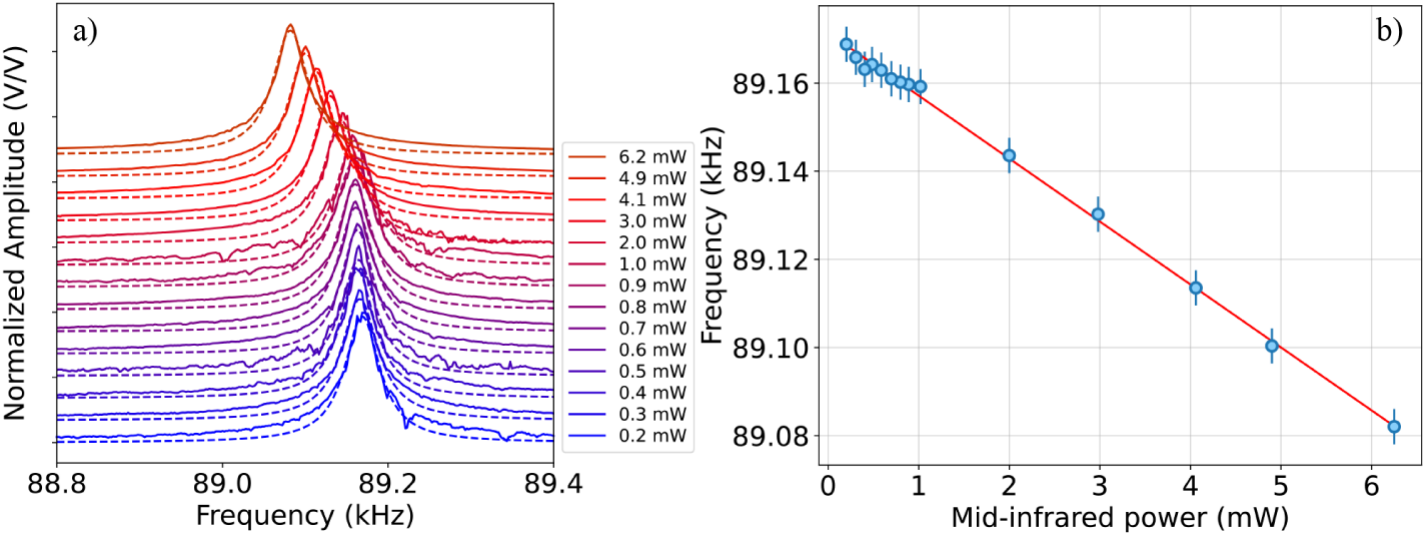
a) Amplitude peak normalized to its maximum value as a function of PZT modulation frequency, plotted at different values of probing mid-IR optical power, when the membrane oscillation is excited by the PZT and no excitation light is sent onto the membrane. Each peak is fit with a Lorentzian function, allowing estimation of the resonance frequency at each impinging power value reported in the legend. b) Membrane resonance frequency, estimated via the Lorentzian fit, as a function of mid-IR probing power. The data (blue points) are fit with a straight line (red curve). The error bar (blue lines) is calculated as the standard deviation of repeated measurements of the resonance frequency for a certain power value.

In both configurations, the impinging power onto the photovoltaic detector has been kept constant, and the detector is operated in the linear responsivity regime as in refs 19 and 20.

## 3 Results and Discussion

At first, the frequency response of the optomechanical system is studied when only the probing radiation, i.e., the QCL’s one, is present and using the PZT to drive the membrane oscillation (*Conf*. 1 in Figure 1). In detail, we studied the resonance frequency shift while varying the QCL power (Figure 2). To this extent, the QCL is kept at a constant working condition (bias current: 470 mA and temperature: 18 °C), and a variable attenuator is used to control the power impinging on the membrane. As shown in Figure 2a, a redshift of the resonance frequency (from 89.169 kHz to 89.082 kHz) occurs due to thermal effects when increasing the mid-IR optical power (from 0.2 mW to 6.2 mW). Indeed, the metal surface of the membrane absorbs the infrared light, resulting in a relaxation of the mechanical stress on the trampoline membrane. For each power value, the resonance frequency is estimated by fitting each resonance peak of Figure 2a with a Lorentzian function. Figure 2b illustrates the linear trend of the frequency shift against the impinging QCL power. While the impinging power is increased, the peak frequency decreases at a rate of (14.28 ± 0.17) Hz/mW, as estimated via linear fit (red line).

After this preliminary characterization, we tested the membrane response when illuminated with both the mid-IR probe beam and the near-IR excitation beam (Figure 3). For this test, the near-IR excitation beam is kept in CW condition (no AM modulation is applied), and the AOM is used simply to vary the average power incident on the membrane, while the PZT is used to map the mechanical resonance under these conditions. The membrane oscillation is indeed driven by the PZT controlled via the lock-in amplifier with a constant-in-amplitude frequency-sweeping signal (*Conf*. 1 in Figure 1). The mid-IR light impinging on the membrane is fixed at its maximum, i.e., 6.2 mW, while the excitation beam power is tuned by changing the amplitude of the AOM RF modulation via the signal generator (see Figure 1). At this probe power level, the feedback signal is strong, and, at the same time, the probe radiation does not saturate the thermal effects on the membrane, as demonstrated by the red-shift of the resonance frequency induced by the near-IR radiation (Figure 3a). Therefore, an independent analysis of the near-IR laser effect on the membrane resonance is possible. As clearly visible in Figure 3a, the resonance peak is red-shifted in frequency when the near-IR laser power increases. Again, for each value of the near-IR impinging power, we estimated the membrane resonance frequency via a Lorentzian fit of the experimental data. The results are shown in Figure 3b. The resonance frequency (blue points) linearly decreases as the near-IR power increases at a rate of (98.8 ± 1.1) Hz/mW, as estimated via a linear fit (red line). We remark that the difference between this rate value and the one related to the QCL power variation (Figure 2) can be explained by different factors: (i) mode matching; (ii) the direction of the incident beams on the membrane; (iii) the use of different windows in the two paths (transmissivity of CaF_2_ windows at 4.5 μm: 95%, transmissivity of COC window at 1.064 μm: 85%); (iv) the presence of a 3-nm Cr layer between the SiN and the 50-nm Au layer affecting (or influencing) the absorption on the 1064 nm beam side.^10^

**Figure 3:**
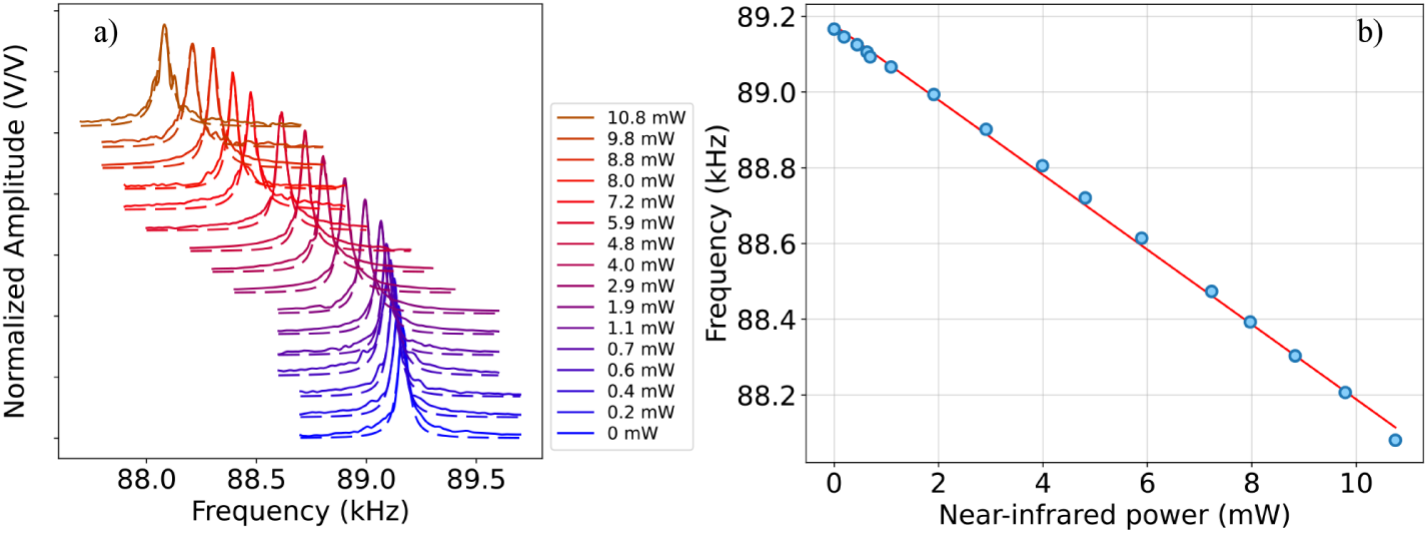
a) Normalized amplitude peak to its maximum value as a function of PZT modulation frequency, plotted at different values of near-IR impinging power, when the membrane oscillation is driven via the PZT and the mid-IR probe beam is kept at a fixed working condition, emitting a power of 6.2 mW. Each peak is fitted via a Lorentzian function, which allows us to estimate the resonance frequency at the peak value for the different values of impinging power. The obtained values are depicted as blue points in graph (b). b) Membrane resonance frequency as a function of the near-IR impinging power. The data (blue points) are fitted via a linear fit (red line). Here, the error bars, obtained as in Figure 2, are not visible.

Further details of the membrane’s resonance stability, noise performance, and sensitivity in *Conf. 1* are provided in Sections 1 and 2 of the Supporting Information.

Finally, we tested the membrane response in *Conf. 2*. Specifically, in this configuration, the AM-modulated near-IR beam induces the membrane oscillation, which is probed via the mid-IR beam kept at constant working conditions with a fixed optical power of 6.2 mW (Figure 4). As described in Section 2, AM modulation of the near-IR light is obtained by externally driving the AOM via the lock-in amplifier. Moreover, to reconstruct the resonance peak, the AM modulation is swept in the frequency range of the membrane resonance. The procedure is repeated for different values of AM modulation amplitude. This allows us to monitor the frequency shift of the membrane resonance peak for different values of the near-IR excitation beam power. As shown in Figure 4a,b, a blue-shift is visible in the peak frequency when increasing the AM modulation amplitude from 100 mV to 500 mV, corresponding to an average near-IR power varying from 9.6 mW to 7.8 mW, respectively. In particular, we can see that, in this case, the frequency shift follows a linear trend when plotted against the impinging near-IR power with a shift rate of (102 ± 4) Hz/mW. Further details of the membrane noise performance and sensitivity in *Conf. 2* are provided in Section 2 of the Supporting Information. In this second configuration, the membrane oscillation, i.e., the presence of the resonance peak in Figure 4, is due to the light-induced force exerted by the near-IR beam. Moreover, the increase in impinging power leads to a redshift of the resonance frequency, as already shown also in *Conf. 1*. In general, the light-induced force might have different contributions, i.e., radiation pressure and photothermal force.^18^ Typically, these two phenomena have, in general, different time scales, i.e., photothermal force is characterized by a slower time scale compared to the quasi-instantaneousness of the radiation pressure.^18^ However, even if their time scales can be quite different, depending on the specific oscillating system (e.g., membrane and substrate materials), the thermal effect—even if dumped—could be non-negligible at resonance frequency.^10,18^ Therefore, depending on the specific system and working conditions, the driving optomechanical force of the membrane oscillation can be dominated by radiation pressure, photothermal force, or a combination of the two. In order to evaluate the photothermal contribution in our system, we first conducted a preliminary characterization of its time scale.

**Figure 4:**
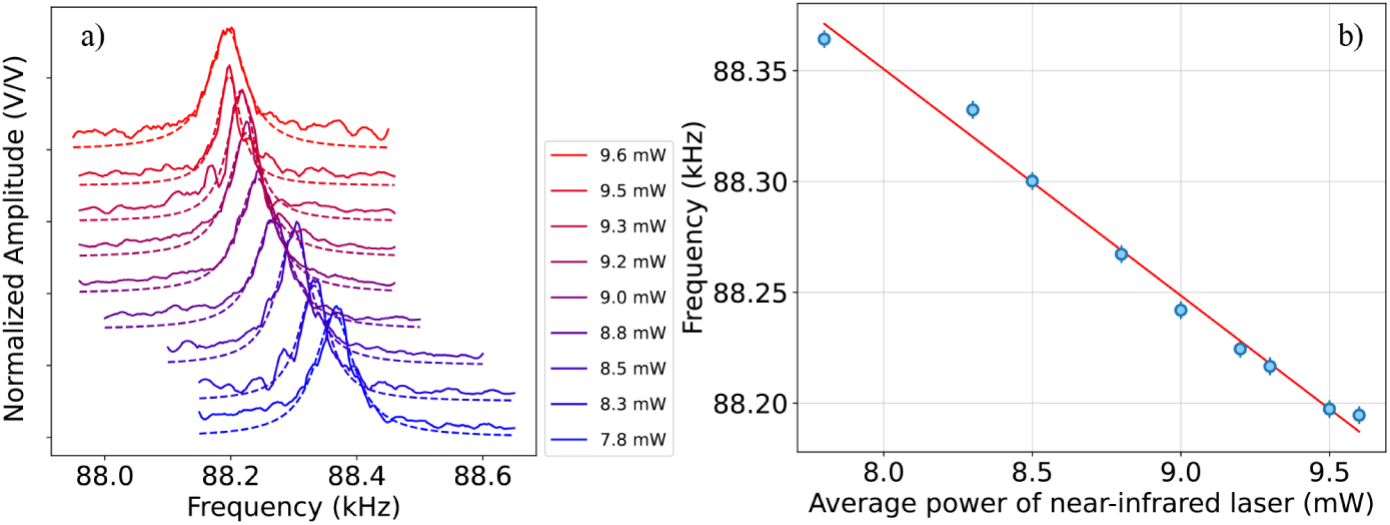
a) Amplitude peak normalized to its maximum value as a function of AM modulation frequency, plotted at different values of AM-modulated near-IR impinging power, when the mid-IR probe beam is kept at a fixed working condition at a power of 6.2 mW. Each peak is fitted via a Lorentzian function, which allows us to estimate the resonance frequency at the peak value for the different values of impinging power. The obtained values are depicted as blue points in graph (b). b) Membrane resonance frequency as a function of near-IR impinging power. The data (blue points) are fit to a straight line (red curve). The error bars are obtained as shown in Figure 2.

Exploiting the setup in *Conf. 1,* we excited the membrane oscillation using the PZT driven with the lock-in amplifier while impinging onto the membrane with both the excitation and probe beams. In addition, we externally applied slow AM modulation to the excitation beam by driving the AOM with the signal generator. Keeping in mind that the piezo frequency sweep is slower (typically a few minutes) than the “slow AM” period, with this measurement, an effective average of the resonance peak is recorded. If the AM modulation is slow enough (i.e., comparable to the thermal relaxation time scale), two peaks are appreciable in the spectrum, as shown in Figure 5. The peak at a higher frequency corresponds to the membrane resonance frequency when illuminated with just the probe radiation (no excitation beam). In contrast, the second peak represents the resonance frequency red-shifted by the thermal effect induced when the membrane is illuminated with both the excitation and probe radiation. In other words, the net effect of AM modulating the excitation beam is frequency modulation of the resonance peak. The spectra are, in fact, characterized by the typical shape of a frequency-modulated signal.^21^ In particular, we studied this effect by varying the frequency of the slow AM modulation from 0.5 Hz up to 1 kHz. In Figure 5, we see that the spectrum consists of two clearly separated peaks up to 20 Hz. Starting from 40 Hz and increasing the modulation frequency, they merge into one single peak. This suggests that the thermal relaxation effect bandwidth is smaller than 40 Hz and much below the membrane resonance frequency (≈90 kHz).

**Figure 5:**
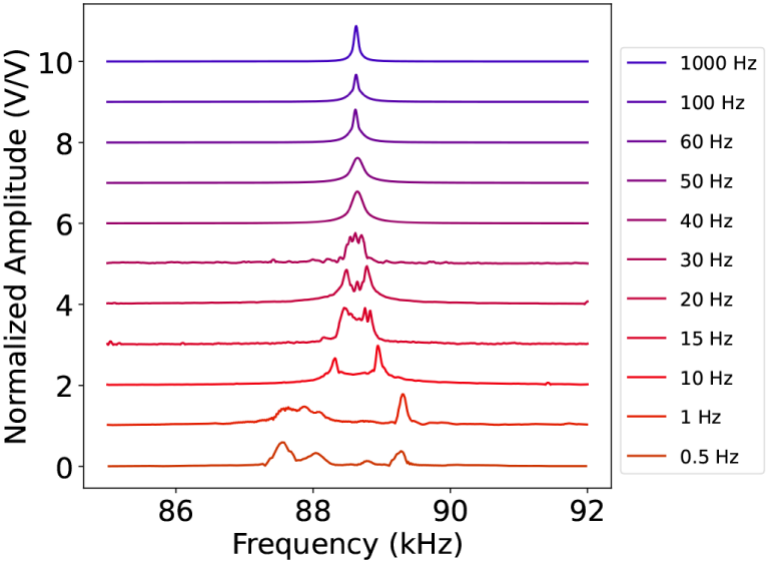
Characterization of the thermal relaxation time scale onto the membrane peak of resonance when the system is driven in *Conf.1* and a slow AM modulation (see the legend) is added to the excitation beam. On the vertical scale, we report the peak amplitude normalized to the maximum, while on the horizontal scale, we report the frequency sweep applied to the PZT.

Therefore, as the photothermal force acts on the system as a low-pass transfer function with a bandwidth below 40 Hz, the photothermal force is attenuated by more than 3 orders of magnitude at resonance (by a factor ≃ 7 × 10^–4^). Nevertheless, with a finite-element simulation, we could retrieve the DC photothermal membrane responsivity as a displacement-per-power coefficient.^10^ Considering the scaling factor due to the thermal transfer function, the photothermal contribution at resonance remains substantially larger than the contribution due to radiation pressure. Using the calculated value of the membrane effective mass^10^ and considering a phenomenologically determined DC photothermal deformation of ∼12 nm/mW, the photothermal force at resonance is estimated to produce an oscillation amplitude of ∼6.67 nm/mW, whereas the oscillation amplitude driven by radiation pressure is approximately ∼0.63 nm/mW. This yields a photothermal-to-radiation-pressure ratio at resonance of about 10.5. This dominant photothermal effect in the system arises from the generated strain in the single-side metal-coated thin-film membrane due to the significant discrepancy in the thermal expansion coefficient between its dielectric and metallic constituent materials under light/thermal excitation. The complete derivation is provided in Section 3 of the Supporting Information.

## 4 Conclusion and Perspectives

This work describes an optomechanical system capable of transferring information from the near-IR to the mid-IR. In detail, an SM-assisted transduction scheme is presented, where a mid-IR QCL is used in a laser-feedback interferometer to probe the oscillation of a membrane when excited via a near-IR beam.

This enables the monitoring of membrane oscillations, optically induced by the near-IR beam, through the SM signal when the beam is amplitude-modulated at frequencies close to the membrane resonance. Therefore, by demonstrating the capability of detecting the resonance signal, we show the potential of using this optomechanical system as a communication gate (i.e., an information transducer) between two different spectral regions. Indeed, the oscillation of the membrane can be used to amplify and transmit the AM modulation signal from the excitation beam to the probe. The tested membrane interface is spectrally broadband, therefore allowing a connection between the mid-IR and the excitation beam, which can be selected over a vast spectral range. Moreover, in principle, this system can also be exploited to control several QCL parameters (e.g., frequency, intensity, phase, or power) via the SM, which boasts a bandwidth close to 100 kHz.

From a technical perspective, this optomechanical platform also offers the possibility to control the QCL emission via self-mixing coupling with an external micromechanical system, leveraging on much faster dynamics compared to bulk optical components. More broadly, by engineering membranes with optimized geometry and properties targeting high-resonance frequencies, the technique presented here could be exploited to create optomechanical interfaces suitable for practical communication applications. In particular, selecting excitation sources at near-IR telecom wavelengths will further enable the development of compact optomechanical interfaces linking fiber-based telecom networks to mid-IR free-space communication channels, particularly suitable for point-to-point^22^ links and atmospheric-resilient transmission.^21–24^ Additionally, membrane arrays fabricated within this architecture could support spatial and frequency multiplexing, either by exploiting the spatial profile of the optical pump or by assigning distinct mechanical resonance frequencies to each element.^25^ Such arrays would expand the system toward advanced sensing and imaging modalities. In particular, the mid-IR QCL self-mixing signal generated by this platform could probe photothermally excited materials at near-IR or visible wavelengths, offering a route toward hybrid photoacoustic mid-IR (or THz) detection schemes.^26^ Collectively, these perspectives highlight the versatility and scalability of the proposed membrane-based optomechanical system for communication, sensing, and imaging applications..

## Supplementary Material



## Data Availability

The data that support the findings of this study are available from the corresponding author upon reasonable request.

## Notes

Additional experimental and data analysis details, including resonance peak stability and sensitivity characterization, and light-induced force simulation (PDF)
