# Supplementary material for: Bridging Mid- and Near-Infrared by Combining Optomechanics and Self-Mixing
Source: ACS Photonics. 2026 Feb 6;13(4):1200–6. doi: 10.1021/acsphotonics.5c03062 (PMC12922770; doi:10.1021/acsphotonics.5c03062)
Supplement: Supplementary file 1 [file ph5c03062_si_001.pdf]

Bridging Mid- and Near-Infrared by Combining Optomechanics and Self-Mixing - *Supporting Information* -

Tecla Gabbrielli,[*,†,‡,△] Chenghong Zhang,[†,‡,△] Francesco Cappelli,[†,‡] Iacopo Galli,[†,‡] Andrea Ottomaniello,[¶] Jérôme Faist,[§] Alessandro Tredicucci,[‖,⊥] Alessandro Pitanti,[‖,#] Paolo De Natale,[†,‡] Simone Borri,[†,‡] and Paolo Vezio[‡,@]

†CNR-INO – Istituto Nazionale di Ottica, Via Carrara, 1 – 50019 Sesto Fiorentino FI, Italy

‡LENS – European Laboratory for Non-Linear Spectroscopy, Via Carrara, 1 – 50019 Sesto Fiorentino FI, Italy

¶Center for Materials Interfaces, Istituto Italiano di Tecnologia, Via R. Piaggio, 34 – 56025 Pontedera, PI, Italy

§Institute for Quantum Electronics, ETH Zürich, 8093, Zürich, Switzerland

‖Dipartimento di Fisica, Università di Pisa, Largo B. Pontecorvo 3, 56127 Pisa, Italy

⊥Laboratorio NEST, CNR – Istituto Nanoscienze, Piazza San Silvestro 12 – 56127 Pisa, Italy

#Laboratorio NEST, CNR – Istituto Nanoscienze, Piazza San Silvestro 12 –56127 Pisa, Italy

@Dipartimento di Fisica e Astronomia e Università di Firenze, Via Sansone 1 – 50019 Sesto Fiorentino FI, Italy

△These authors equally contributed to this work.

E-mail: tecla.gabbrielli@ino.cnr.it

1 Resonance peak stability and reproducibility

As a preliminary characterization of the system response and its reproducibility, we estimate the stability of membrane resonance by taking multiple measurements, repeated in time, of the resonance peak while keeping the amount of impinging power constant while operating the system in *Conf. 1*, i.e., using the piezo to drive the oscillation.

Fig. S1(a) reports the stability of the membrane resonance when just the mid-IR probe beam impinges on it (no excitation beam). In detail, the impinging mid-IR power is fixed at 6.2 mW and the measurement of the resonance peak is repeated 17 times. The standard deviation of the extracted resonance frequencies is 4 Hz. Analogously, 10 traces of the membrane's resonance are used to estimate the resonance stability when both the mid-IR probe beam (impinging power fixed at 6.2 mW) and the near-IR excitation beam impinge on the membrane (impinging power fixed at 5.2 mW). The results are shown in Fig. S1(b). The standard deviation of the extracted resonance frequencies is 3 Hz.

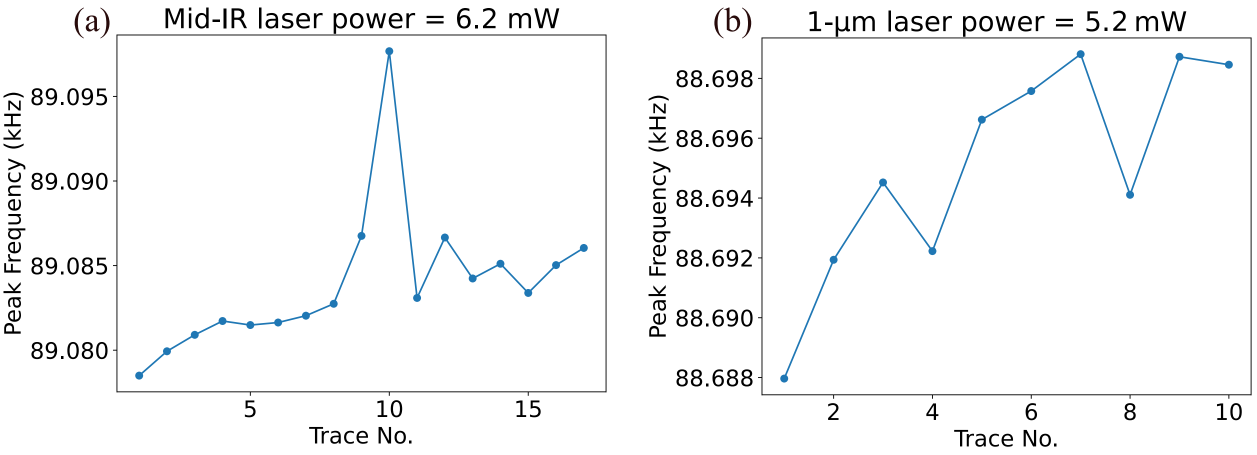

Figure S1: Stability of the membrane's resonance peak frequency when oscillated by the PZT. (a) 17 resonance traces obtained in *Conf. 1* with a fixed incident mid-infrared power of 6.2 mW, showing a standard deviation of 4 Hz. (b) 10 resonance traces obtained in *Conf. 1*, with a fixed incident mid-IR power of 6.2 mW and a fixed near-IR of 5.2 mW, showing a standard deviation of 3 Hz.

2 Baseline characterization and sensitivity estimation

The baseline of the resonance peak is an important parameter that might set a lower limit in terms of minimum signal detectability and the self-mixing detection dynamic range. Fig. S2 shows the baseline signal as the impinging power varies. In detail, the reported values associated with each power value are extracted by averaging the baseline signal within a 20-point window on the resonance tail. In our measurements, each trace is acquired with the same integration time (10 ms). For each dataset shown in Fig. S2, the average window has been set at a fixed relative distance (as far as possible) from the resonance peak frequency. In detail, Fig. S2(a) shows the baseline signal

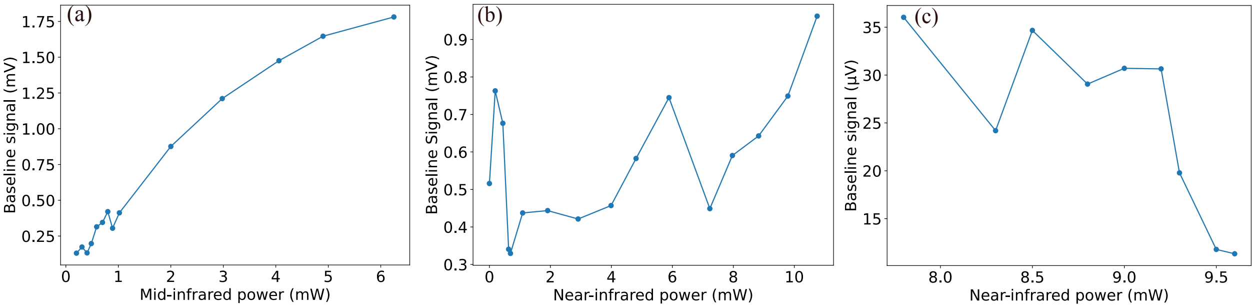

Figure S2: Baseline signals under different operating conditions. The baseline signal is calculated by averaging 20 data points in the resonance tails (as far as possible from the resonance frequency) for the dataset (i.e., traces) acquired under three different conditions: (a) varying mid-IR laser power in *Conf. 1* (membrane oscillation driven via PZT) when no excitation light is sent onto the membrane, (b) varying near-IR laser power in *Conf. 1* (membrane oscillation driven via PZT) at a fixed mid-IR power of 6.2 mW, and (c) varying near-IR laser power in *Conf. 2* (membrane oscillation optically induced by the AM modulated excitation beam) at a fixed mid-IR power of 6.2 mW.

when the system operated in *Conf. 1*, i.e., oscillation is driven by the PZT, and just the mid-IR probe laser impinges on the membrane. The laser working condition (i.e., temperature and bias current) is kept constant, and the impinging power varies via a variable attenuator. In this case, as the probe laser power increases, so does the back-reflected signal coupled in the laser waveguide, and a clear increasing trend is visible in the baseline signal when the mid-IR incident power increases. Instead, when the mid-IR impinging power is kept constant (6.2 mW) and the near-IR excitation light is added in the experimental setup, a clear trend cannot be identified whenever the membrane is oscillated via the PZT (Fig. S2(b), system operated in *Conf. 1*) or via the light-induced force by

the AM-modulated excitation radiation (Fig. S2(c), system operated in *Conf. 2*). Moreover, it is possible to notice that the baseline signal is significantly lower in the case of *Conf. 2* (Fig. S2(c)), where the baseline signal is of the order of tens of μV signals in contrast to the mV level measured in *Conf. 1* (Figs. S2(a) and (b)).

To better clarify the role of the baseline in the two different configurations explored in this work (*Conf. 1* and *Conf. 2*), we estimate the sensitivity of our system by calculating the fluctuation associated with the baseline to understand the minimum signal amplitude detectable on top of the baseline signal. In Fig. S3, we report the signal-to-noise ratio (SNR) obtained as the ratio between the peak amplitude (obtained by subtracting the baseline signal estimated via the procedure shown in Fig. S2 from the peak maximum) and the standard deviation of the baseline in the windows declared in the analysis related to Fig. S3. For this analysis, we remark that, as our work is a proof of concept, the setup is not equipped with fine control of the optical path length, which would allow for fine-tuning of the phase match and maximization of the SM interference amplitude. In future work, we plan to optimize the performance of the setup via a full characterization and control of all its parameters for practical sensing and communication applications. Nevertheless, even at this early stage, we attempted to obtain the maximum possible value during the measurements, given the limitations of the current setup. The same three different experimental conditions explored in Fig. S2 are also reported in the case of Fig. S3: plot (a) shows the SNR at varying the mid-IR power, when the system is operated in *Conf. 1* and just the mid-IR probe laser impinges on the membrane; plot (b) reports the SNR as a function of the near-infrared power when the system is operated in *Conf. 1* and both the mid-IR probe (power fixed at 6.2 mW) and the near-IR excitation beam impinge on the membrane; finally, plot (c) shows the SNR as a function of the near-infrared power when the system is operated in *Conf. 2* and the mid-IR power is kept constant at 6.2 mW. From this analysis, we can see that the sensitivity, i.e. the SNR is higher in *Conf. 1*, suggesting a lower sensitivity in *Conf. 2*. Once again, this is a preliminary characterization. In view of future applications, a better control of experimental parameters, such as the optical path fine-tuning, and a refined analysis and selection of optimal working conditions for both the self-mixing source and

excitation beam could help reduce noise in *Conf. 2*.

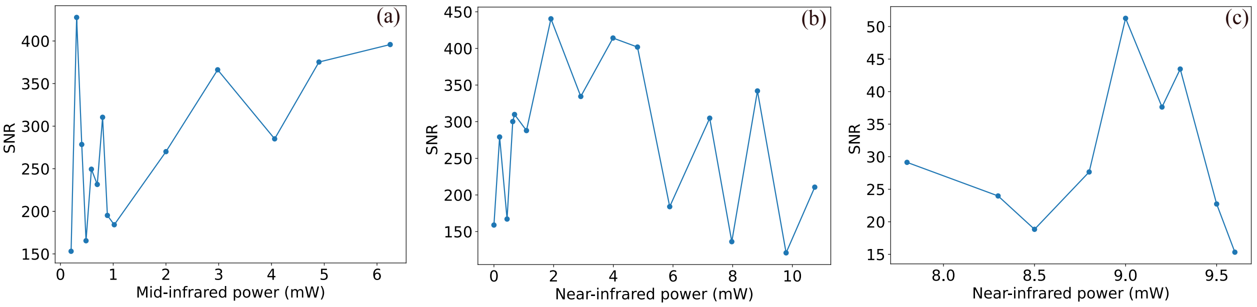

Figure S3: SNR as a function of impinging power under different operation conditions. The SNR is calculated as the ratio between the amplitude peak and the corresponding baseline signal as estimated in Fig. S2. The reported SNR datasets correspond to the conditions described in Fig. S2: (a) varying mid-IR laser power in *Conf. 1* (membrane oscillation driven via PZT) when no excitation light is sent onto the membrane, (b) varying near-IR laser power in *Conf. 1* (membrane oscillation driven via PZT) at a fixed mid-IR power of 6.2 mW, and (c) varying near-IR laser power in *Conf. 2* (membrane oscillation optically induced by the AM-modulated excitation beam) at a fixed mid-IR power of 6.2 mW.

3 Membrane oscillation induced by photothermal and radiation pressure

As already stated in the main text, the light-induced force exerted by the AM-modulated excitation radiation onto the membrane has two main contributions, i.e., the photothermal force and the radiation pressure. Here, we report a toy model that allows us to independently estimate the membrane displacement generated by each mechanism and, so, to quantitatively estimate the strength of these two contributions. The mechanical susceptibility of a resonator (i.e. the membrane in our setup) is given by:

$$\chi(\omega) = \frac{1}{m_{\text{eff}}(\omega_{\text{m}}{}^2 - \omega^2 + i\omega_{\text{m}}\omega/Q)}, \tag{1}$$

where m_{eff} is the effective mass, ω_m is the mechanical resonance angular frequency, and Q is the quality factor. Consequently, the susceptibility at resonance frequency simplifies to:

$$|\chi(\omega_{\text{m}})| = \frac{Q}{m_{\text{eff}}\omega_{\text{m}}^2}. \tag{2}$$

The membrane displacement driven by radiation pressure at resonance frequency is equal to:

$$X_{\mathrm{rp}}(\omega_{\mathrm{m}}) = \alpha_{\mathrm{rp}} \frac{P_{\mathrm{mod}}}{m_{\mathrm{eff}}\omega_{\mathrm{m}}^2} Q, \tag{3}$$

where P_{mod} is the modulated optical power amplitude and $\alpha_{\mathrm{rp}} = 2\beta/c$ is the radiation-pressure coupling coefficient (i.e. radiation pressure per unit of power) with β representing the fraction of light effectively transferring momentum to the membrane, and c is the speed of light. In the following dissertation, we assume $\beta = 1$.

When estimating the photothermal displacement of the membrane induced by the AM near-infrared excitation beam, we first consider the steady-state photothermal response generated by a constant-amplitude incident beam. The static photothermal deflection X_{DC} given by a constant incident power P_{DC} can be written as:

$$X_{\mathrm{DC}} = \chi(0)\alpha_{\mathrm{th}} P_{\mathrm{DC}}, \tag{4}$$

where $\chi(0) = 1/(m_{\mathrm{eff}}\omega_{\mathrm{m}}^2)$ is the static mechanical susceptibility obtained from (3) with $\omega \to 0$. We can therefore estimate the photothermal force-per-power coefficient α_{th}:

$$\alpha_{\mathrm{th}} = \frac{X_{\mathrm{DC}}}{\chi(0) P_{\mathrm{DC}}}. \tag{5}$$

We can then obtain the frequency-dependent photothermal force amplitude by applying the low-pass filter function $H_{\mathrm{th}}(\omega) = 1/(1+i\omega\tau)$ to include the thermal response,[1] where τ is the thermal response time of the membrane resonator. A sinusoidal AM modulated power of amplitude P_{mod} yields to the photothermal force amplitude:

$$F_{\mathrm{th}}(\omega) = \alpha_{\mathrm{th}} P_{\mathrm{mod}} H_{\mathrm{th}}(\omega). \tag{6}$$

The related resonant photothermal displacement is thus:

$$X_{\mathrm{th}}(\omega_{\mathrm{m}}) = |\chi(\omega_{\mathrm{m}})|\, \alpha_{\mathrm{th}}\, P_{\mathrm{mod}}\, |H_{\mathrm{th}}(\omega_{\mathrm{m}})|\,. \tag{7}$$

Using the DC calibration (Eq. (5)), this expression reduces to:

$$X_{\mathrm{th}}(\omega_{\mathrm{m}}) = \eta\, X_{\mathrm{DC}}\, \frac{1}{\sqrt{1+(\omega_{\mathrm{m}}\tau)^2}}\, Q\,, \tag{8}$$

where $\eta = P_{\mathrm{mod}}/P_{\mathrm{DC}}$ is the fractional modulation depth. For a numerical evaluation, we use the following estimated values: $m_{\mathrm{eff}} = 4.16\times10^{-11}$ kg, $Q = 1400$, and $X_{\mathrm{DC}} = 12$ nm/mW as quantified from the Finite Element Method (FEM) simulation results reported in Ref .[2] From the experimental results of this work, we set the resonance frequency at $f_{\mathrm{m}} = 90\,\mathrm{kHz}$, and the frequency cutoff of the thermal effect to $f_{\mathrm{th}} = 40\,\mathrm{Hz}$ ($\tau = 1/(2\pi f_{\mathrm{th}}) \simeq 4$ ms). representative AM modula-

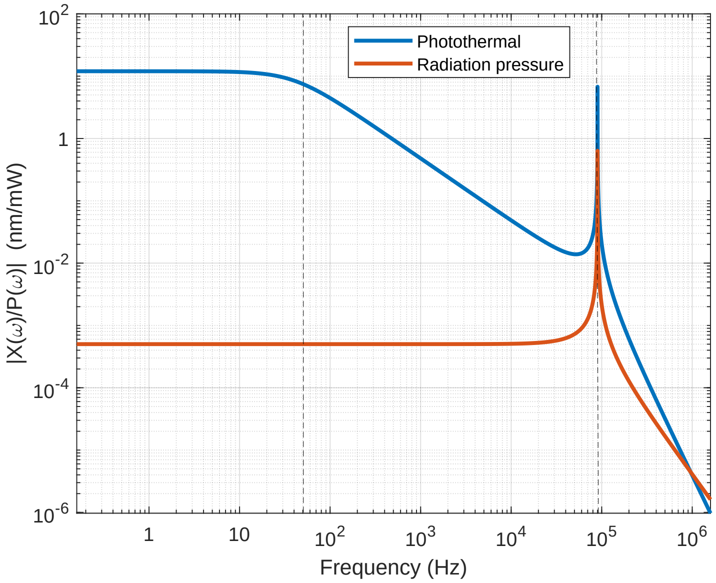

Figure S4: Oscillation amplitude spectrum for the photothermal force (blue curve) and radiation pressure (red curve). Vertical dashed lines represent $f_{\mathrm{th}} \simeq 4$ ms and $f_{\mathrm{m}} = 90\,\mathrm{kHz}$.

tion with an average power $P_{\mathrm{avg}} = 10\,\mathrm{mW}$ and a modulation depth of 20% (i.e. $P_{\mathrm{mod}} = 2\,\mathrm{mW}$), the displacements induced by the two different effects are quantitatively estimated at the resonance $f_m = 90\,\mathrm{kHz}$ as $X_{\mathrm{th}} \approx 14.9\,\mathrm{nm}$ and $X_{\mathrm{rp}} \approx 1.4\,\mathrm{nm}$, giving a total displacement of $X_{\mathrm{tot}} \approx 16.3\,\mathrm{nm}$.

Thus, in this representative case, the radiation pressure accounts for roughly $\sim$ 8.6% of the combined resonant deformation in this representative case while the photothermal effect provides the remaining $\sim$ 91.4%, confirming that photothermal forces dominate the membrane excitation. The oscillation amplitude spectra induced by the photothermal force and radiation pressure are shown in Fig. S4.

References

(1) Kanellopulos, K.; Ladinig, F.; Emminger, S.; Martini, P.; West, R. G.; Schmid, S. Comparative analysis of nanomechanical resonators: sensitivity, response time, and practical considerations in photothermal sensing. *Microsystems & Nanoengineering* **2025**, *11*, 28.

(2) Vezio, P.; Ottomaniello, A.; Vicarelli, L.; Salih, M.; Li, L.; Linfield, E.; Dean, P.; Mattoli, V.; Pitanti, A.; Tredicucci, A. Membrane-Mediated Conversion of Near-Infrared Amplitude Modulation into the Self-Mixing Signal of a Terahertz Quantum Cascade Laser. *Photonics* **2025**, *12*.
